# Myeloid 12/15-LOX regulates B cell numbers and innate immune antibody levels *in vivo*

**DOI:** 10.12688/wellcomeopenres.10308.1

**Published:** 2017-01-04

**Authors:** Sarah N. Lauder, Victoria J Tyrrell, Keith Allen-Redpath, Maceler Aldrovandi, David Gray, Peter Collins, Simon A Jones, Philip R Taylor, Valerie O'Donnell

**Affiliations:** 1 Systems Immunity Research Institute, Cardiff University, Cardiff, UK; 2 Institute of Infection & Immunity, Cardiff University, Cardiff, UK; 3 Institute of Immunology and Infection Research, University of Edinburgh, Edinburgh, UK

**Keywords:** lipoxygenase, B cells, inflammation

## Abstract

*Background*. The myeloid enzyme 12/15-lipoxygenase (LOX), which generates bioactive oxidized lipids, has been implicated in numerous inflammatory diseases, with several studies demonstrating an improvement in pathology in mice lacking the enzyme. However, the ability of 12/15-LOX to directly regulate B cell function has not been studied.

*Methods.* The influence of 12/15-LOX on B cell phenotype and function, and IgM generation, was compared using wildtype (WT) and 12/15-LOX ( *Alox15* ^-/-^) deficient mice. The proliferative and functional capacity of splenic CD19 ^+^ B cells was measured *in vitro* in response to various toll-like receptor agonists.

*Results*. WT and *Alox15* ^-/-^ displayed comparable responses. However *in vivo*, splenic B cell numbers were significantly elevated in *Alox15* ^-/-^ mice with a corresponding elevation in titres of total IgM in lung, gut and serum, and lower serum IgM directed against the 12/15-LOX product, 12-hydroxyeicosatetraenoic acid-phosphatidylethanolamine (HETE-PE).

*Discussion.* Myeloid 12/15-LOX can regulate B cell numbers and innate immune antibody levels *in vivo*, potentially contributing to its ability to regulate inflammatory disease. Furthermore, the alterations seen in 12/15-LOX deficiency likely result from changes in the equilibrium of the immune system that develop from birth. Further studies in disease models are warranted to elucidate the contribution of 12/15-LOX mediated alterations in B cell numbers and innate immune antibody generation to driving inflammation *in vivo*.

## Introduction

Lipoxygenases (LOX) are immunoregulatory enzymes that oxidise polyunsaturated fatty acids, forming bioactive mediators, including leukotrienes, eicosanoids and oxidized phospholipids (oxPL). The leukocyte-type 15-LOX is expressed by reticulocytes, IL-4/IL-13 stimulated monocytes, eosinophils and airway epithelia ^1^. The murine homolog, 12/15-LOX, is highly expressed by peritoneal macrophages and elevated in murine atheroma. In multiple studies, this isoform has been found to play a complex role in controlling inflammatory disease. Mice lacking 12/15-LOX are resistant to atherosclerosis, hypertension and diabetes, but experience greater inflammatory responses in arthritis ^2– 7^. During acute inflammation, such as in bacterial peritonitis, 12/15-LOX supports macrophage and monocyte recruitment ^8^. Furthermore, it is required for effective apoptotic cell clearance during peritoneal inflammation, maintaining tolerance to self-antigens ^9^. In atherosclerosis, 12/15-LOX orchestrates oxidation of LDL, promoting foam cell formation and plaque development, and enhancing endothelial cell inflammation ^2, 10^.

It has been proposed that 12/15-LOX is critical for functional haematopoiesis in mice, with its deletion causing spontaneous myeloproliferative disease (MPD) characteristic of chronic myeloid leukaemia (CML) ^11^. However, this has not reproduced in other colonies of 12/15-LOX deficient mice, despite mild splenomegaly being observed ^12– 14^. Importantly, while the effect of 12/15-LOX deficiency on the myeloid compartment of the hematopoietic system has been widely studied, its potential regulation of the lymphoid lineage is unknown.

B cells participate in resolution of inflammation and infection, in both innate and adaptive responses. Residing in the periphery and spleen, B-1 cells produce ‘Natural IgM’ that contributes a significant proportion of the circulating IgM present in naïve, unchallenged mice. This recognises evolutionarily-conserved self and non-self-antigens expressed by pathogens and apoptotic cells. The transfer of B-1 derived IgM can halt development of autoimmunity, and IgM titres are elevated in atheroprotected mice ^15, 16^. IgG is secreted by B2 cells that reside predominantly in the spleen and secondary lymphoid organs. These constitute the largest population of B cells in the mouse. B2 cells form part of the host’s adaptive immune response, producing high affinity, antigen-specific IgG subclasses. B2 cells have been shown, through adoptive transfer, to induce a pro-atherogenic state in mice, the mechanism of which is not fully understood, but is independent of IgG ^17^. Elevated IgG is associated with pathogenicity in a number of autoimmune conditions including SLE, rheumatoid arthritis and Sjögren’s syndrome ^18, 19^. Of relevance to this study, either the absence of 12/15-LOX or increase in IgM secreting B1a cells is atheroprotective, but a functional link between these two observations has not been established ^2, 20^. The observation that increased lipid oxidation-specific epitope-reactive IgM elicits protection against atherosclerosis suggests that B cells and their antibodies, including those that recognise oxidized lipids, mediate a key role in preventing development of chronic inflammatory pathology ^21^.

Given the known role of B cells in atherosclerosis and other diseases, in which 12/15-LOX is known to play a pro-inflammatory role, we decided to undertake a full characterisation of the B cell compartment in healthy mice lacking the enzyme. Our studies found that the proportions of splenic B cell populations are skewed in favour of innate IgM producing B cells, consistent with mild spenomegaly and the known generation of protective antibodies in inflammation. In contrast, there was no functional defect in B cells from these mice *in vitro* or *in vivo,* indicating that their ability to respond to inflammatory activation is preserved. The present data indicate an effect of the enzyme on B cell physiology that may contribute to its known role in regulating inflammatory disease.

## Methods

### Mice

Male and female C57BL/6 (WT) mice were purchased from Charles River UK at 8 weeks (numbers are provided on figure legends for specific experiments), and aged and sex-matched with *Alox15* deficient mice (12/15-LOX ^-/-^), bred at the Heath Park Campus Animal Facility, Joint Biological Services in-house. Animals were housed in specific pathogen free conditions in groups between 4 and 8 animals per cage. Mice had access to standard mouse chow and water *ad libitium* and were maintained on 12h light:12h dark cycles. Mice were sacrificed at 24–26 weeks of age using Schedule 1 methods, in compliance with UK Home Office Regulations (PPL 30/3150).

### Immunoglobin ELISAs

Mouse IgA, IgG and IgM ELISAs (eBioscience) were used to determine the different immunoglobin titres in serum, peritoneal lavage, gut lavage and bronchoalveolar lavage (BAL).

### Flow cytometry and FACS analysis

For all experiments, cells were analysed by flow cytometry (FACSCanto II, Becton-Dickinson, CA, USA) and the data analysed using FlowJo software version 10 (Treestar, Ashland, OR, USA). Rat anti-CD16/CD32 was obtained from BD Pharmingen (San Jose, CA, USA) and used as directed in the datasheet. All flow cytometry protocols included an Fc Block step.

Detailed information on antibodies used are as follows (all antibodies were used as per the datasheets provided by the manufacturers):

Pacific Blue rat anti-mouse CD19 antibody. Clone: 6DS. Product number: 115523 (Biolegend) ^22^.

Brilliant Violet 510 rat anti-mouse/human CD45R/B220 antibody. Clone: RA3-6B2. Product number: 103247 (Biolegend) ^23^.

APC-rat anti-mouse CD5 antibody. Clone: 53-7.3. Product number: 100626 (Biolegend) ^24^.

APC rat anti-mouse CD21/CD35 (CR2/CR1) antibody. Clone: 7E9. Product number: 123412 (Biolegend) ^25^.

PerCP/Cy5.5 rat anti-mouse I-A/I-E antibody. Clone: MS/114.15.2. Product number: 107625 (Biolegend) ^26^.

PE/Cy7 rat anti-mouse CD86 antibody. Clone: GL1. Product number: 105014 (Biolegend) ^27^.

APC-Cy7 rat anti-mouse CD62L antibody. Clone: MEL14. Product number: 104428 (Biolegend) ^28^.

APC rat anti-mouse CD40 antibody. Clone: 3/23. Product number: 124612. Used as directed in the datasheet (Biolegend) ^29^.

FITC rat anti-mouse/human CD44 antibody. Clone: IM7. Product number: 103006 (Biolegend) ^30^.

Rat anti-mouse IgM PE-Cyanine 7 antibody. Clone: 11/41. Product number: 25-5790-82 (eBioscience) ^31^.

Rat anti-mouse CD93 PerCP-Cyanine 5.5 antibody. Clone: AA4.1. Product number: 45-5892-82 (eBioscience) ^32^.

Rat anti-Mouse CD43 FITC antibody. Clone: eBio R2/60. Product number: 11-0431-85 (eBioscience) ^33^.

Rat anti-mouse CD16/CD32 antibody. Clone: 2.4G2. Product number: 553142 (BD Pharmingen) ^34^.

### Isolation of B cell subsets

B cell subsets were isolated from spleens of 24–26 week old mice. Splenic CD19 ^+^ B cells were purified by positive MACS microbead selection (Miltenyi Biotec, Bergisch-Gladbach, Germany), according to the manufacturer’s instructions. Follicular (Fo) and marginal zone (Mz) B cells were isolated from the spleens using a Marginal Zone and Follicular B cell isolation kit (Miltenyi Biotec). Splenic B1a B cells were purified by positive selection using a B1a cell isolation kit (Miltenyi Biotec). B cells were cultured at 2 × 10 ^6^ cells/ml in complete IMDM media, supplemented with 5% (v:v) FCS and 0.1% (v;v) insulin-transferrin-selenium.

### B cell activation studies

Purified B cell subsets were stimulated with Toll-like receptor (TLR) ligands for 16 hours at 37ºC. Lipopolysaccharide (LPS; TLR4 ligand) from *E. Coli* 0:111 B4 (Sigma Aldrich, St. Louis, MO, USA) was used at 10 µg/ml, loxoribine (TLR7 ligand; Source Bioscience, Nottingham, UK) was used at 100 µM, and CpG (ODN 1826; TLR9 ligand; Source Bioscience) used at 100 µg/ml. Following stimulation, changes in B cell activation markers CD40, CD44, CD62L and CD86 were determined by flow cytometry.

### B cell proliferation studies

In total, 2 x 10 ^5^ B cells were cultured with 10 ng/ml IL-4 (R&D Systems) and 10 µg/ml CD40 (Biolegend) ± 10 µg/ml LPS in 96-well round bottom plates. Cells were incubated for 72 or 96 hours at 37°C, with [ ^3^H]-thymidine (1µCi/well; Perkin Elmer, Waltham, MA, USA) added for the final 18 hours. Cell proliferation was assessed by [ ^3^H]-thymidine incorporation. The stimulation index was calculated by dividing the mean counts per minute (cpm) of stimulated cells by the mean cpm of unstimulated cells.

### Determination of circulating antibodies to hydroxyeicosatetraenoic acide-phosphatideylethanolamines (HETE-PEs) in mice

Specific antibody titers to individual HETE-PEs (12-, 5- and 15- isomers) were determined with a chemiluminescent ELISA, as previously described ^15^. Briefly, lipids generated in house were coated onto Microfluor plates at 20 µg/ml PBS and subsequently blocked with 0.5% (w:v) fish-gelatin in 0.27mM PBS-EDTA. Lipid synthesis is provided in ref ^35^. Serum from WT or 12/15-LOX ^-/-^ male and female mice at 24–26 weeks of age was diluted in 0.27mM PBS-EDTA (1:12) and incubated for 1 hour at room temperature. Bound IgM was measured using an anti-mouse IgM alkaline phosphatase-conjugated secondary antibody (goat anti-mouse IgM-alkaline phosphatase, polyclonal, product number: A9688, Sigma-Aldrich. Ab was titrated in-house and used at a dilution of 1:40,000) and Lumi-Phos (Lumigen, Southfield, MI, USA). Data is expressed as relative light units in 100 ms (RLU/100ms).

### Statistical analysis

Graphpad Prism Version 5 was used for all statistical analysis. All statistical assessments used the Mann-Whitney U test. P values of ≤ 0.05 were considered significant (*), with values of ≤ 0.01 considered highly significant (**).

## Results

### IgM titres are elevated in the absence of 12/15-LOX

**Figure 1.**
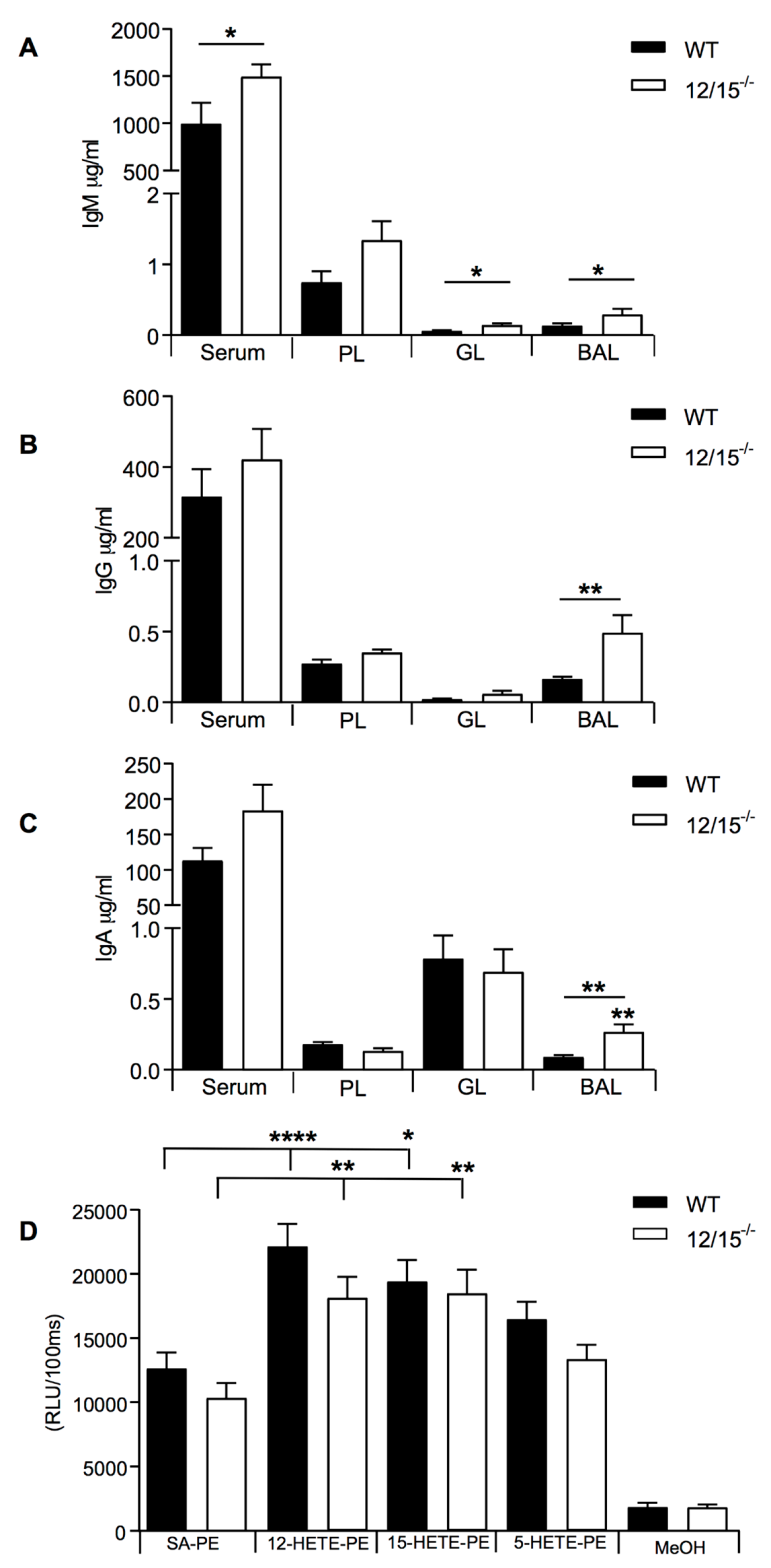
Global IgM titres and BAL IgA and IgG are elevated in 12/15-LOX deficient mice. ( **A**). *IgM titres are elevated in 12/15-LOX ^-/-^ mice:* Total IgM was determined in the serum, peritoneal (PL), gut (GL) and broncoalveolar (BAL) lavage of WT and 12/15-LOX ^-/-^ mice (n = 17 serum & PL; n = 14 GL; n = 8 BAL). ( **B** and **C**) *Total IgG and IgA titres were significantly elevated in BAL of 12/15-LOX ^-/-^ mice:* Total IgG ( **B**) and IgA ( **C**) was determined in the serum, PL, GL and BAL of WT and 12/15-LOX ^-/-^ mice (n = 17 serum & PL; n = 14 GL; n = 8 BAL). Mean ± SEM, Mann Whitney U test, *p ≤ 0.05, **p ≤ 0.01. ( **D**) *IgM directed against hydroxyeicosatetraenoic acid-phosphatidylethanolamines (HETE-PsE) are significantly elevated in serum:* Titers of IgM antibodies to 15-HETE-PE, 12-HETE-PE and 5-HETE-PE were determined by diluting serum 1:12 and testing binding to the indicated antigens as described in Methods. 1-stearoyl-2-arachidonyl-PE (SA-PE) was used as unoxidised control (n = 40 – 50). Statistical significance was determined using one-way ANOVA with Tukey post-hoc test, *p ≤ 0.05, **p ≤ 0.01, ***p ≤ 0.001.

Total IgM titres were significantly increased in serum (p ≤ 0.05), gut (p ≤ 0.05) and lung (p ≤ 0.05), and was elevated but not significantly in peritoneal lavages of mice deficient in 12/15-LOX as compared to WT. ( Figure 1A). Similar elevations in IgA (p ≤ 0.01) or IgG (p ≤ 0.01) were also seen in lung lavage of 12/15-LOX ^-/-^ mice, although there was a general trend towards elevated IgG in other fluids ( Figure 1B and C). Macrophages and eosinophils generate oxidized phospholipids termed 12-hydroxyeicosatetraenoic acid-phosphatidylethanolamines (12-HETE-PEs) via 12/15-LOX along with lower levels of the 15-HETE-PE positional isomers ^36^. Thus, we sought to determine whether loss of the enzyme resulted in altered IgM immunoreactivity towards its lipid products. Immunoreactivity towards the neutrophil-derived 5-HETE-PE generated by 5-LOX was also tested ^37^. Significantly increased IgM titres against 12- (p ≤ 0.001, or 0.01 for WT or 12/15-LOX ^-/-^, respectively) or 15-HETE-PE (p ≤ 0.05 or 0.01, for WT or 12/15-LOX ^-/-^, respectively) versus the non-oxidized 1-stearoyl-2-arachidonyl-PE (SAPE) were detected in both 12/15-LOX ^-/-^ and wild type serum, indicating that lipid oxidation leads to an IgM response *in vivo* ( Figure 1D). There was reduced IgM recognition in serum from 12/15-LOX ^-/-^ mice versus wild type against 12-HETE-PEs, although this trend did not reach statistical significance ( Figure 1D).

### Splenic B cell subsets associated with IgM secretion are elevated in 12/15-LOX deficiency

**Figure 2.**
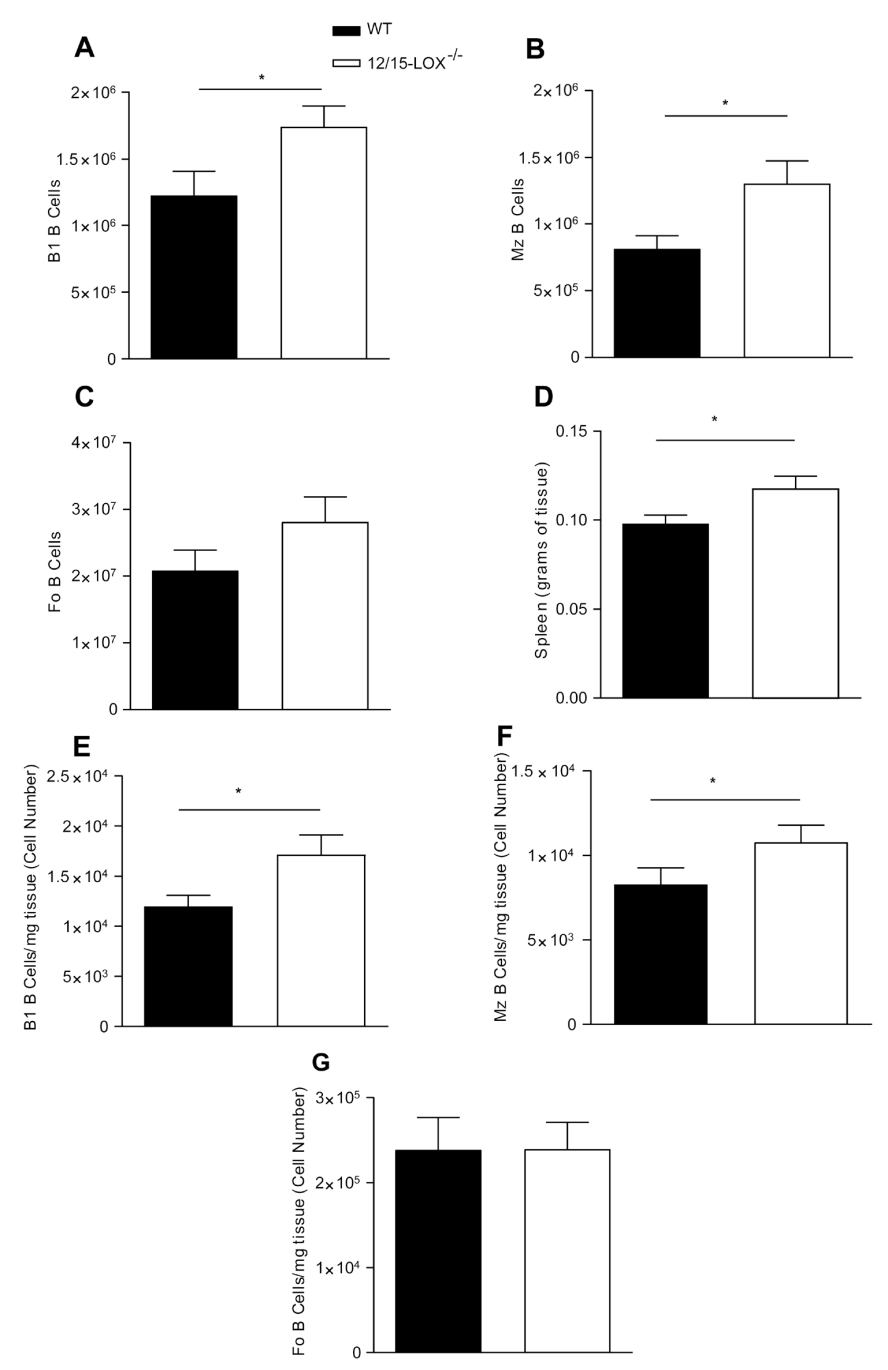
12/15-LOX deficiency is associated with increased spleen B cells. ( **A**) *Spleen B1 cells are significantly increased in 12/15-LOX ^-/-^ mice:* Total numbers of B1 (CD19 ^+^, B220 ^+^, IgM ^+^, CD23 ^-^, CD93 ^-^, CD43 ^+^) cells per spleen were determined in WT and 12/15-LOX ^-/-^ mice (n = 14 – 17). ( **B**) *Spleen Marginal zone (Mz) cells are significantly elevated in 12/15-LOX ^-/-^ mice:* Total numbers of Mz (CD19 ^+^, B220 ^+^, IgM ^+^, CD23 ^-^, CD93 ^-^, CD21/35 ^+^) cells per spleen were determined in WT and 12/15-LOX ^-/-^ mice (n = 14 – 17). ( **C**) *Spleen Follicular (Fo) B cells are significantly elevated in 12/15-LOX ^-/-^ mice:* Total numbers of Fo B cells (CD19 ^+^, B220 ^+^, IgM ^+^, CD23 ^+^, CD93 ^-^) cells per spleen were determined in WT and 12/15-LOX ^-/-^ mice (n = 14 – 17). ( **D**) *12/15-LOX ^-/-^ mice have larger spleens:* Spleen wet weight was determined (n = 14 – 17 per group). Mann Whitney U test, *p ≤ 0.05. ( **E**) Total numbers of B1 (CD19 ^+^, B220 ^+^, IgM ^+^, CD23 ^-^, CD93 ^-^, CD43 ^+^) cells per mg spleen ( **F**) Mz (CD19 ^+^, B220 ^+^, IgM ^+^, CD23 ^-^, CD93 ^-^, CD21/35 ^+^) and ( **G**) Fo B cells (CD19 ^+^, B220 ^+^, IgM ^+^, CD23 ^+^, CD93 ^-^) per mg of tissue were determined from the spleens of WT and 12/15 ^-/-^ mice at 26 weeks of age (n = 14 – 17).

B cells from WT and 12/15-LOX ^-/-^ mice spleens were examined using flow cytometry. A significant elevation in B1 and Marginal Zone (Mz) B cells was observed in 12/15-LOX ^-/-^ mice (p ≤ 0.05) with a trend towards higher Follicular (Fo) B cell numbers per spleen ( Figure 2A–C). 12/15-LOX ^-/-^ mice develop mild splenomegaly with ageing, and this was reproduced herein (p ≤ 0.05) ( Figure 2D) ^11, 14^. Interestingly, we found that the increased B1 and Mz B cells numbers observed in 12/15-LOX ^-/-^ mice were independent of spleen size as indicated by a higher number of cells per mg tissue ( Figure 2E–G).

### 12/15 LOX deficiency does not affect B cell populations in the periphery

**Figure 3.**
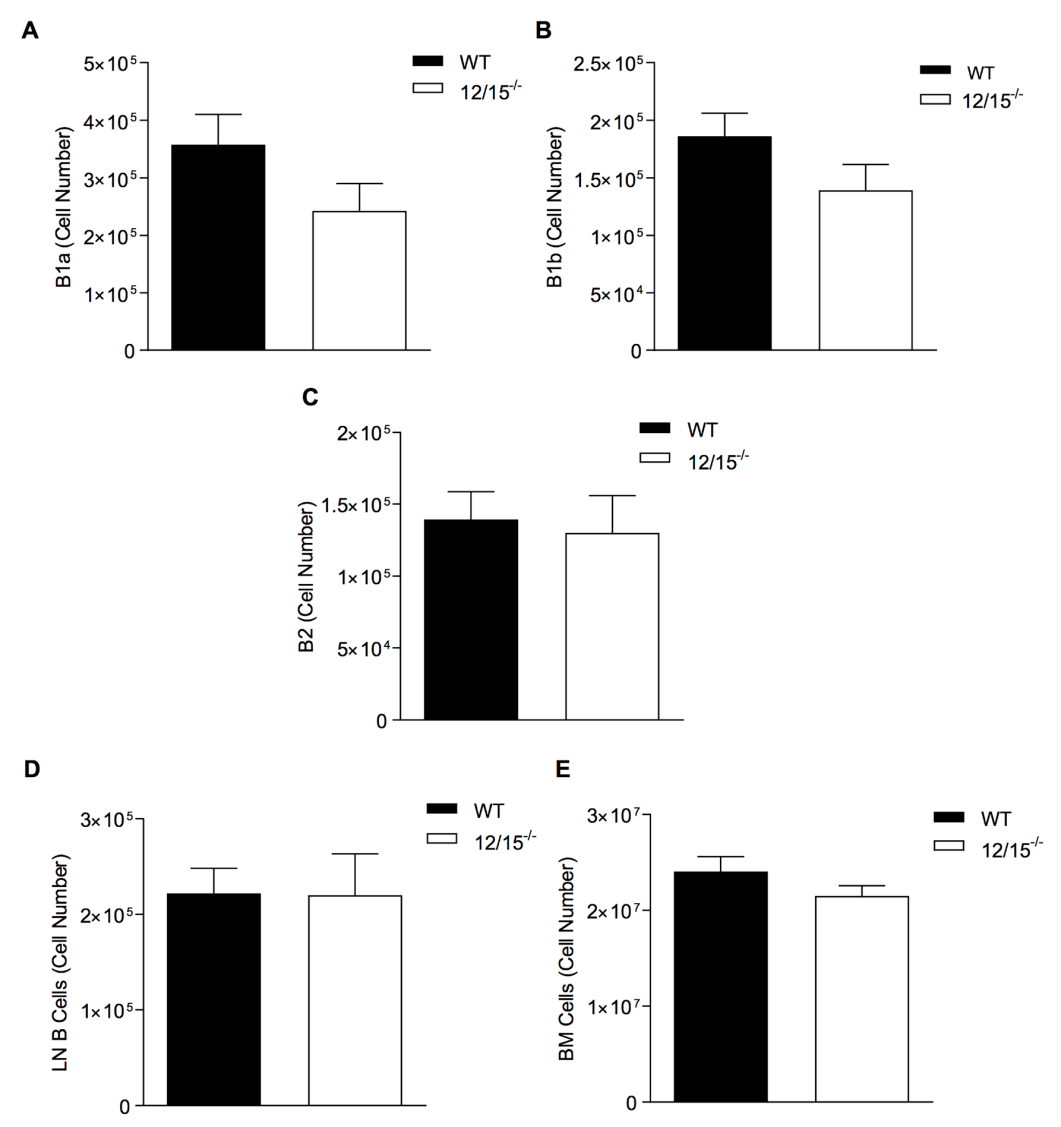
12/15-LOX deficiency has little effect on numbers of resident B cell. ( **A**) *B1a cell numbers are not altered in 12/15-LOX deficiency:* Total numbers of B1a (CD19 ^+^, IgM ^+^, CD43 ^+^, CD23 ^-^, CD5 ^+^) cells in the peritoneal cavity of WT and 12/15-LOX ^-/-^ mice were determined by flow cytometry (n = 14 – 16 per group). ( **B**) *B1b cell numbers are not altered in 12/15-LOX deficiency:* Total numbers of B1b (CD19 ^+^, IgM ^+^, CD43 ^+^, CD23 ^-^, CD5 ^-^) cells in the peritoneal cavity of WT and 12/15-LOX ^-/-^ mice were determined by flow cytometry (n = 14 – 16 per group). ( **C**) *B2 cell numbers are not altered in 12/15-LOX deficiency:* Total numbers of B2 cells (CD19 ^+^, IgM ^+^, CD43 ^-^, CD23 ^+^, CD5 ^-^) in the peritoneal cavity of WT and 12/15-LOX ^-/-^ mice were determined by flow cytometry (n = 14 - 16/group). ( **D**) *Lymph node (LN) cell numbers are unaltered by 12/15-LOX deficiency:* Total numbers of B cells in the inguinal LN (CD19 ^+^, B220 ^+^, CD23 ^+^, CD93 ^-^, CD21/35 ^+^, n = 14 – 17/group). ( **E**) *Bone marrow (BM) cells in the femur are not altered by 12/15-LOX deficiency:* The total number of BM cells present in the femur of WT and 12/15 ^-/-^ mice (n = 14 – 17/group).

To determine reasons for the elevated IgM seen in 12/15-LOX deficiency, we examined the frequency of various B cell populations in the periphery of these animals. There was a trend towards lower numbers of peritoneal B1a and B1b cells, but this was not significant, while B2 cell numbers were unchanged ( Figure 3A–C). Similar numbers of CD19 ^+^ B cells were found in the inguinal lymph nodes or bone marrow of WT and 12/15-LOX ^-/-^ mice ( Figure 3D). Conflicting studies have been published regarding the occurrence of myeloproliferative disease (MPD) in 12/15-LOX deficiency ^11– 14^. Herein, WT and 12/15-LOX ^-/-^ mice bone marrow contained a comparable number of total cells in the femur, indicating that these animals are not suffering from MPD ( Figure 3E).

### B cells from naïve animals show a comparable activation threshold following TLR engagement

**Figure 4.**
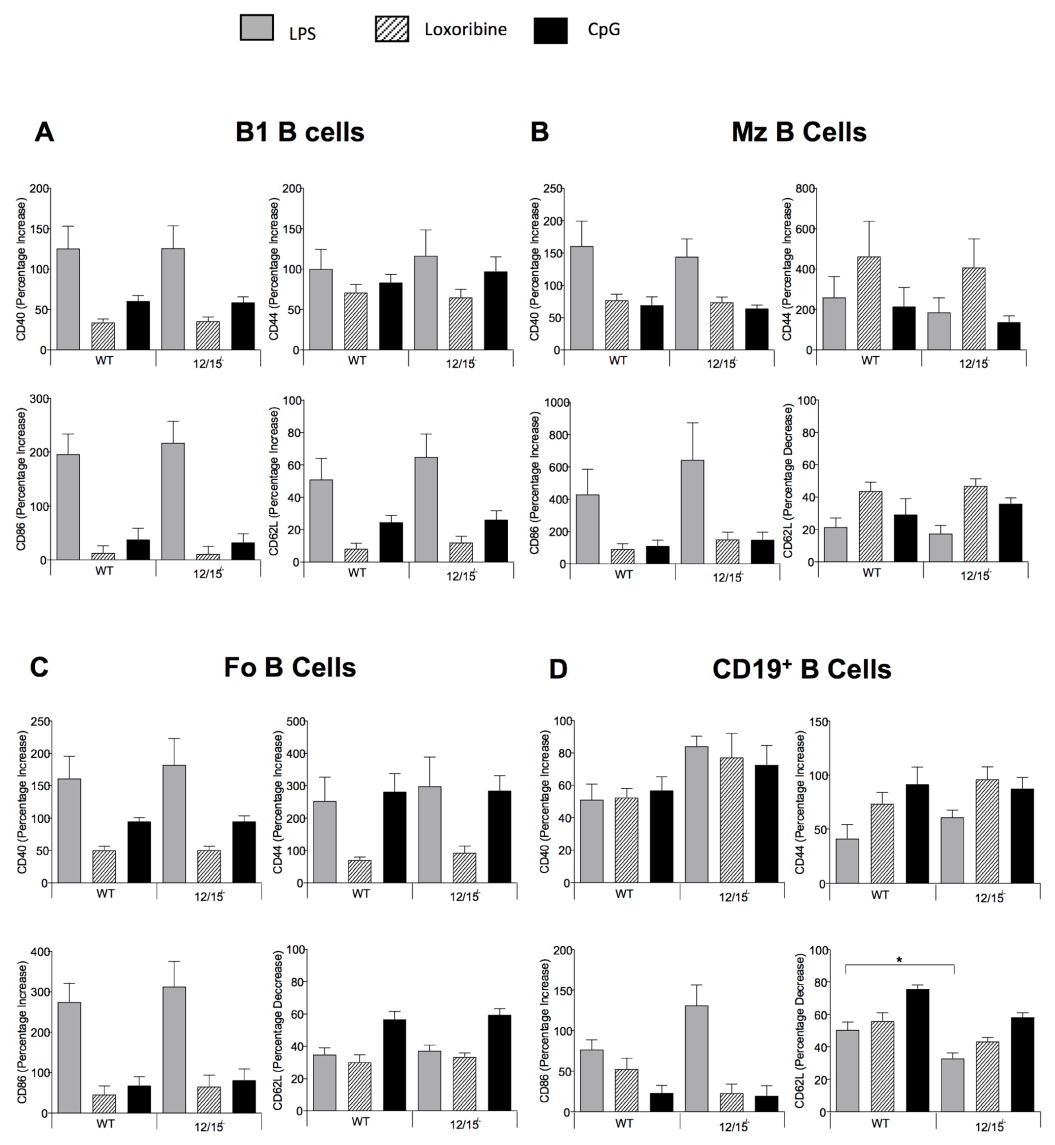
TLR mediated activation of B cells is not modified by 12/15-LOX deficiency. *( **A**)* *Splenic B1 B cells exhibit comparable activation thresholds in response to TLR mediated stimulation:* Splenic B1 cells were isolated as described in the Methods and cultured overnight with the TLR agonist indicated. Flow cytometric analysis of the activation markers CD40, CD44, CD86 and CD62L as indicated, was determined and the percentage increase or decrease determined from unstimulated B1 cells cultured overnight. *( **B**)* *Splenic Marginal zone (Mz) B cells exhibit comparable activation thresholds in response to TLR mediated stimulation:* Splenic Mz cells were isolated and cultured overnight with the TLR agonist indicated. Flow cytometric analysis was undertaken as in ( **A**). *( **C**) Splenic Follicular (Fo) B cells exhibit comparable activation thresholds in response to TLR mediated stimulation:* Splenic Fo cells were isolated and cultured overnight with the TLR agonist indicated. Flow cytometric analysis was undertaken as in ( **A**). *( **D**)* *Splenic Total CD19 ^+^ B cells exhibit comparable activation thresholds in response to TLR mediated stimulation:* Splenic CD19 ^+^ B cells were isolated and cultured overnight with the TLR agonist indicated. Flow cytometric analysis was undertaken as in ( **A**). For ( **A–D**), data is displayed as mean ± SEM, n = 19 per group from three combined independent experiments, one-way ANOVA, with Tukey post-hoc comparison, *p ≤ 0.05.

Mixed CD19 ^+^ B cells and purified B1, Mz and Fo B cells were isolated from the spleens of 26 week old mice and activated overnight using TLR agonists. Surface activation markers were determined using flow cytometry. There was a marked difference in the response to TLR agonists between the different B cell populations. B1 cells were most responsive to LPS stimulation for all activation markers tested ( Figure 4A). Mz B cells favoured either LPS or Loxoribine, whilst Fo B cells preferred LPS or CPG, depending upon the activation marker analysed ( Figure 4B and C). Mixed population CD19 ^+^ B cells were comparable to Fo B cells favouring LPS or CpG stimulation ( Figure 4D). The TLR differential upregulation of activation markers in B cell subsets was comparable with previous studies ^38^. For all B cell populations tested, there were no significant differences observed between WT and 12/15-LOX ^-/-^ B cells with respect to expression of surface activation markers, under these activation conditions. However, total CD19 ^+^ B cells derived from 12/15 ^-/-^ mice had altered activation thresholds in comparison to WT, with significantly reduced CD62L expression following LPS stimulation (p ≤ 0.05), similarly Loxoribine and CpG also induced reduced CD62L expression in comparison to WT CD19 ^+^ cells. Conversely, the expression of CD40 was notably increased with all TLR agonists in 12/15-LOX ^-/-^ CD19 ^+^ B cells.

### Splenic B cell proliferation is not increased by 12/15-LOX deficiency

**Figure 5.**
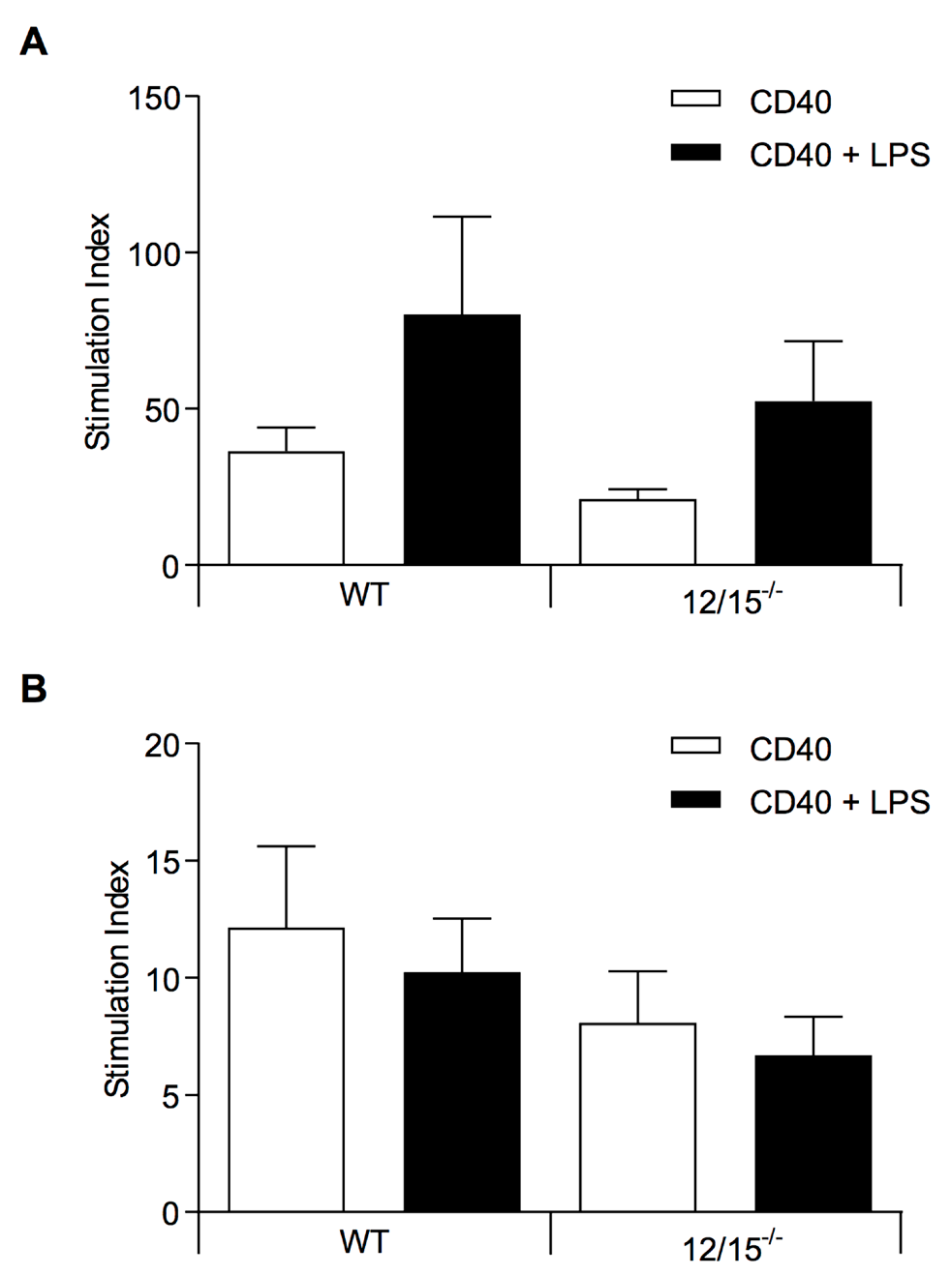
B cell proliferation is unaffected by 12/15-LOX deletion. Splenic CD19 ^+^ B cells isolated from WT and 12/15 ^-/-^ mice at 26 weeks of age were stimulated with CD40 ± LPS as described in the Methods. Proliferation was measured by thymidine incorporation after 72 hours ( ***A***) and 96 hours ( ***B***). Data is expressed as the mean Stimulation Index ± SEM, n = 13–14 per group from two combined independent experiments.

Since spleen size and total numbers of B1 and Mz B cells are elevated in 12/15-LOX ^-/-^ mice, we sought to determine the proliferative capacity of splenic B cells. Using splenic CD19 ^+^ B cells we found that B cell proliferation in response to either CD40 ligation alone or in combination with LPS was slightly, but not significantly, lower in cells from 12/15-LOX ^-/-^ mice ( Figure 5). Proliferation of B cell populations from either strain following LPS stimulation was considerably greater than CD40 activation alone at day 3 ( Figure 5A), although this effect had diminished when proliferation was compared at day 4 post stimulation ( Figure 5B). Thus, elevated B cell proliferation does not explain increased spleen size in 12/15-LOX ^-/-^ mice.

## Discussion

Herein, we show that mice lacking the leukocyte type 12/15-LOX, primarily expressed by myeloid cells *in vivo*, display subtle changes in B cell biology, specifically elevated splenic B cell numbers in concert with larger spleens, and systemically elevated IgM ( Figure 1 and Figure 2). Elevated B cells were not seen in the periphery, indicating this to be spleen specific ( Figure 3). IgG and IgA were also elevated in BAL, suggesting that 12/15-LOX-expressing eosinophils and lung resident macrophages may modulate B cell activation status in this compartment ( Figure 1). 12/15-LOX is not expressed by B cells, thus its expression by other cell types may subtly modify the balance between B cells of an innate (B1 and Mz) or an acquired phenotype (Fo and B2). Indeed it is known that the absence of 12/15-LOX attenuates airway allergic inflammation and remodelling *in vivo* ^39^.

Mild splenomegaly in the absence of 12/15-LOX was previously reported by us and others, and confirmed herein ^11, 14^. However, increased splenic B1 and Mz B cell numbers were independent of increased organ size, suggesting they make up a greater proportion of spleen cells ( Figure 3). One possible explanation is decreased removal of B cells via apoptotic clearance by 12/15-LOX expressing splenic macrophages. In this regard, an essential role for oxidized phospholipids from this enzyme in sequestering soluble MFG-E8, which supports apoptotic cell clearance of resident peritoneal macrophages, was previously reported ^9^. B cell activation *in vitro* was unaltered in the present study indicating that the cells are functionally normal in the absence of 12/15-LOX. This further supports the idea that alterations in B cell number and IgM levels in mice are likely mediated via interactions with other 12/15-LOX expressing cells, such as macrophages.

Murine serum contained IgM that selectively recognised oxidized phospholipids generated by 12/15- or 12-LOX isoforms expressed by macrophages, eosinophils and platelets, but not 5-HETE-PE generated by neutrophil 5-LOX ( Figure 1D). Interestingly, IgM recognition of HETE-PEs was partially reduced in 12/15-LOX ^-/-^ mice versus wild type ( Figure 1D). While not reaching statistical significance, this indicates that the 12/15-LOX contributes at least in part to generation of an IgM response to oxidized phospholipids. We note that mice also generate 12-HETE-PE via platelet 12-LOX, thus a complete loss of immunoreactivity towards this lipid from a single gene knockout would not be expected. 

Several studies to date have demonstrated that elevated IgM is atheroprotective in ApoE ^-/-^ mice ^15– 17^. Circulating immunoglobulin titres were determined in the serum and a number of peripheral locations in WT and 12/15-LOX ^-/-^ mice by the present study. We have seen that 12/15-LOX ^-/-^ mice have elevated circulating IgM titres and increased numbers of splenic B1 cells responsible for IgM secretion. We propose that this increased IgM titre may afford early protection against the development of atherosclerosis. Thus, during plaque formation, resulting from high fat feeding, in 12/15 ^+/+^ animals, greater oxidation of phospholipids occurs, which promotes atherosclerotic development through increased lipid peroxidation and endothelial inflammation and foam cell formation ^10^. Furthermore, the altered apoptotic cell clearance by 12/15 ^-/-^ macrophages ^9^ may result in the development of autoantibodies that result in greater clearance of cells expressing self-antigens associated with oxidation of LDL, which prevent the development of plaque *in vivo*.

The 12/15-LOX, which is primarily expressed by myeloid cells, is already known to modulate inflammatory disease in multiple cell types. This study shows that the absence of this enzyme can also modulate B cell biology *in vivo*. Our study extends the known role of 12/15-LOX into regulation of B cells, suggesting additional potential modulatory actions in inflammatory disease where it already plays a known role ^3, 5– 7^. With respect to this study we have demonstrated that during the steady state 12/15-LOX appears to be vital for maintaining tolerance, in the absence of the enzyme the B cell balance *in vivo* becomes skewed. We speculate that the mild splenomegaly observed suggests the development of pre-clinical autoimmunity, as evidenced by the generation of autoantibodies ^7, 40^. A role of 12/15-LOX has been postulated for several autoimmune conditions, including rheumatoid arthritis, systemic lupus erythromatoses and antiphospholipid syndrome ^7^. Therefore, a full characterisation of the role of 12/15-LOX in driving B cell-dependent development and progression of these diseases is warranted.

## Data availability

All raw data from the manuscript have been submitted to Open Science Framework, doi: 10.17605/OSF.IO/2BQ74 ^41^ ( https://osf.io/2bq74/).
