# Supplementary material for: Myeloid 12/15-LOX regulates B cell numbers and innate immune antibody levels in vivo
Source: Wellcome Open Res. 2017 Jan 4;2:1. [Version 1] doi: 10.12688/wellcomeopenres.10308.1 (PMC5321417; doi:10.12688/wellcomeopenres.10308.1)
Supplement: Peer review history

# Peer review: Referee response for version 1

**DOI:** 10.21956/wellcomeopenres.11101.r19393

**Published:** 2017-02-14

**Authors:** Stephen G. Ward

**Affiliations:** 1 Department of Pharmacy and Pharmacology, University of Bath, Bath, UK

The manuscript by Lauder *et al *investigates the role of 12-15-lipoxygenase in B cell phenotype and function using healthy mice deficient in the enzyme (Alox15-/- mice).  They report that in the 12/15-Lox deficient mice there are (i) increased global titres of IgM  as well as increased IgA and IgG in the lung; (ii) increased splenic B cell subsets associated with IgM secretion, but no effect on numbers of peripheral or splenic B cells; (iii) comparable B cell activation thresholds following TLR engagement. The changes in IgM, IgA and IgG were for the most part, very subtle. 

 While largely descriptive, the work is interesting, well performed and the data is well generally described and interpreted.   The authors conclude that since 12/15-LOX is not expressed by B cells, its expression by other cell types likely leads to regulation of B cell subsets.  The notion that oxidised lipids that can regulate B cell subsets and antibody production (albeit modestly), is interesting.  Although, the work provides no mechanistic insight into how this occurs, the authors do provide some speculative explanation based on published observations from other groups.   Personally, I would of found a schematic of the model being proposed quite helpful in this respect.

 The B cell activation studies were performed with single concentrations of TLR agonists at single time-points.  There is no indication as to why these concentrations were chosen and whether these are sub-optimal or optimal concentrations. More detailed analysis of kinetics and agonist concentration vs response relationships may reveal differences in kinetics and sensitivity to the TLR agonists.

I have read this submission. I believe that I have an appropriate level of expertise to confirm that it is of an acceptable scientific standard.

# Peer review: Referee response for version 1

**DOI:** 10.21956/wellcomeopenres.11101.r19556

**Published:** 2017-01-23

**Authors:** Coleen A. McNamara, Hema Kothari, Prasad Srikakulapu

**Affiliations:** 1 Department of Medicine, Robert M. Berne Cardiovascular Research Center, University of Virginia, Charlottesville, VA, USA; 2 University of Virginia, Charlottesville, VA, USA

The study by Lauder *et al*. utilized mice globally deficient in 12/15-LOX and demonstrated an increase in splenic B1 and MZ cell populations and serum and gut lavage (GL) and bronchoalveolar lavage (BAL) IgM. There is also increased BAL IgG and IgA. The finding that enzymes that mediate lipid oxidation may regulate B cell subsets and antibody production is of interest and the paper is well-written. 

 One of the main findings is that while there is no statistically significant difference in any B cell subset in the peritoneal cavity and Fo B cells in the spleen, there is a modest increase in B1 and MZ B cells in the spleens of 12/15-LOX ^-/-^ mice.  Quantifying the number of cells in a particular B cell subsets is dependent on the gating strategy used to define these subsets.  B cell gating is complex and expression of surface markers used to identify different B cell subsets are context dependent.  Since B1 cells express high levels of CD19 and lower levels of B220 than B2 cells in all tissues ^1^^-^^3^, gating on CD19 ^hi^ B220 ^low^ seems more appropriate for B1 cell quantification.

 In the spleen B1 gating, the authors did not include the CD19 ^hi^ B220 ^low^, so likely did not count all the B1 cells.  In addition, CD43 is expressed on B1 cells but not B2 cells. However, in the last plot of the B1 spleen gating strategy (raw data in supplement), the authors appear to have included both CD43 ^low/-^ and CD43 ^+^ cell in the B1 cell gate.  An FMO or isotype control could help separate CD43+ from CD43- and ensure that they are not including non B1 cells.

  In the peritoneal cavity, in the legend to Figs 3A and 3B, it is mentioned that B1a and B1b cells were determined as CD19 ^+^, IgM ^+^, CD43 ^+^, CD23 ^-^ and CD5 ^+^ or Cd5 ^-^. However, CD23 is not included in the PerC gating strategy for B1a or B1b cells in the supplemental raw data. In the gating for B1b cells, the authors use CD11b+.  While most of the B1 cells in the PerC are CD11b ^+^, some B1 cells are CD11b ^-^^4^. Therefore, separating out the CD5- population in terms of CD11b does not further discriminate B1b cells and may even eliminate some.  The gating in PerC using CD43 in the raw data for B1 and B2 identification seems to show that CD43 is not a good discriminator in this location as a good number of the CD43- cells are CD23- (raw data of Fo gating in PerC).

 Was live/dead staining performed? If yes, it would strengthen the study to include it in all gating schemes.

**Additional comments and suggestions to consider:**

 Kinder *et al*. ^5^ have demonstrated that Alox15 mice exhibit reduced lymphocytes and monocytes in peripheral blood. Percentage of B220+ B cells in the spleen and number of B cell progenitors in the bone marrow were significantly reduced in the Alox15 mice as compared to the wild-type mice. However, in the present study, the authors found an increase in B1 and marginal zone B cells in the spleen of 12/15-LOX ^-/-^. What is the possible reason for the difference in the results? Did the authors analyze B cell subsets in the blood and bone marrow? Could these mice be different in some other way.  It would help if the authors could provide details on Alox15 deficient mice used in this study and compare them to those used in the Kinder studies.

 As other cells including eosinophils, adipocytes, epithelial cells also express 12/15-LOX, and the studies were performed using a global knockout, the observed differences may not be due to myeloid-specific expression of 12/15-LOX.

 It would strengthen the proposed links between the enzyme and biological outcomes if the authors could show levels of 12/15-LOX-mediated lipid metabolites (12-HETE and 15-HETE) and 5-HETE in the serum of wild-type and 12/15-LOX ^-/-^ mice.

 The conclusion in the abstract that there was “lower serum IgM directed against 12/15-LOX product, 12-HETE-PE” seems overstated as the small reduction in IgM against 12-HETE-PE did not achieve statistical significance even with an n of 40-50.

 The authors have depicted the percentage change in expression of surface activation markers compared to unstimulated cells. Do the graphs represent change in cell percentage or change in mean fluorescence intensity for different activation markers? The plotted values and flow histogram data do not exactly match. For example, for CD86 expression, the histograms show increase in percentage of positive cells or cell surface expression in stimulated cells as compared to the unstimulated cells for both genotypes. However, the plotted graph shows percent increase in CD86 in LPS treated cells while decrease with loxoribine and CpG treatment considering the unstimulated was 100%. It would be good to see a direct comparison between the wild-type and 12/15-LOX ^-/-^ cells not as a percentage of unstimulated.  i.e. Was there a difference in expression of these markers on various B cell subsets between wild-type and 12/15-LOX ^-/-^ under unstimulated/basal conditions?

**Minor points:**

1. While it is correct in the result section, in figure 2C legend, spleen FO cells are said to be “ significantly” elevated in 12/15-LOX ^-/-^ mice, although this is not statistically significant.
2. The raw data file for 12/15-LOX ^-/-^splenic Fo B cells activation marker strategy is mislabeled as WT splenic Fo B cells.
3. Since CD5 was not used to differentiate between B1a and B1b in the spleen gating raw data, this is only B1 gating strategy not B1a.

We have read this submission. We believe that we have an appropriate level of expertise to confirm that it is of an acceptable scientific standard, however we have significant reservations, as outlined above.

## References

[1] DemariaOPagniPPTraubSde GassartABranzkNMurphyAJValenzuelaDMYancopoulosGDFlavellRAAlexopoulouL: TLR8 deficiency leads to autoimmunity in mice. *J Clin Invest*.2010;120(10) : 10.1172/JCI42081 3651-62 10.1172/JCI42081 20811154PMC2947223

[2] RosenfeldSMPerryHMGonenAProhaskaTASrikakulapuPGrewalSDasDMcSkimmingCTaylorAMTsimikasSBenderTPWitztumJLMcNamaraCA: B-1b Cells Secrete Atheroprotective IgM and Attenuate Atherosclerosis. *Circ Res*.2015;117(3) : 10.1161/CIRCRESAHA.117.306044 e28-39 10.1161/CIRCRESAHA.117.306044 26082558PMC4527334

[3] HarmonDBSrikakulapuPKaplanJLOldhamSNMcSkimmingCGarmeyJCPerryHMKirbyJLProhaskaTAGonenAHallowellPSchirmerBTsimikasSTaylorAMWitztumJLMcNamaraCA: Protective Role for B-1b B Cells and IgM in Obesity-Associated Inflammation, Glucose Intolerance, and Insulin Resistance. *Arterioscler Thromb Vasc Biol*.2016;36(4) : 10.1161/ATVBAHA.116.307166 682-91 10.1161/ATVBAHA.116.307166 26868208PMC4808436

[4] GhosnEEYangYTungJHerzenbergLAHerzenbergLA: CD11b expression distinguishes sequential stages of peritoneal B-1 development. *Proc Natl Acad Sci U S A*.2008;105(13) : 10.1073/pnas.0712350105 5195-200 10.1073/pnas.0712350105 18375763PMC2278228

[5] KinderMWeiCShelatSGKunduMZhaoLBlairIAPuréE: Hematopoietic stem cell function requires 12/15-lipoxygenase-dependent fatty acid metabolism. *Blood*.2010;115(24) : 10.1182/blood-2009-09-243139 5012-22 10.1182/blood-2009-09-243139 20357242PMC2890149

# Peer review: Referee response for version 1

**DOI:** 10.21956/wellcomeopenres.11101.r19157

**Published:** 2017-01-20

**Authors:** Andrew P. Sage

**Affiliations:** 1 Department of Medicine, University of Cambridge, Cambridge, UK

Lauder *et al* present novel data characterising the B cell compartment in 12/15 lipoxygenase deficient mice. They find that 12/15LOX-/- mice have enhanced serum IgM levels as well as increased IgM, IgA and IgG in the lung. They provide data to show that increased IgM derives from increased levels of spleen B1 and marginal zone (IgM producing) B cell subsets, but that these cells do not display fundamental intrinsic changes in activation thresholds or proliferation. Since B cells do not themselves express 12/15LOX, the phenotype may depend on 12/15LOX functions in macrophages or eosinophils.

 The data and methods are carefully and accurately described, and the data is interpreted with balance and justification.

 I have the following minor comments:

**Methods**: A more detailed description, or reference to such, of the broncoalveolar lavage and gut lavage protocols, would help readers to be able to fully reproduce this data.

**Study design:** It is possible that there are minor differences in commensal microbes between mice purchased from Charles River and those bred in house. It is conceivable that this could influence microbe reactive B1 and MZ B cells. Therefore, a more robust comparison would be littermate control mice.

 The doses of TLR ligands used are high (especially CpG). It is possible that any differences might have been missed.

 One flow cytometry subset missing that would add further insight to the link between IgM and B1/MZ B cells is plasma cell numbers (CD138+ B220- IgM+ or -) in spleen and bone marrow.

**Discussion: **The authors should consider discussing Baldan *et al* ^1^ that shows a potentially similar phenotype of lung Ig levels in ABCG1-/- mice, since 12/15LOX and ABCG1 may be linked ^2^.

 12/15LOX mice have previously been evaluated in a model of atherosclerosis ^3^.

I have read this submission. I believe that I have an appropriate level of expertise to confirm that it is of an acceptable scientific standard.

## References

[1] BaldanAGonenAChoungCQueXMarquartTJHernandezIBjorkhemIFordDAWitztumJLTarlingEJ: ABCG1 is required for pulmonary B-1 B cell and natural antibody homeostasis. *J Immunol*.2014;193(11) : 10.4049/jimmunol.1400606 5637-48 10.4049/jimmunol.1400606 25339664PMC4239162

[2] NagelinMHSrinivasanSNadlerJLHedrickCC: Murine 12/15-lipoxygenase regulates ATP-binding cassette transporter G1 protein degradation through p38- and JNK2-dependent pathways. *J Biol Chem*.2009;284(45) : 10.1074/jbc.M109.028910 31303-14 10.1074/jbc.M109.028910 19713213PMC2781528

[3] RongSCaoQLiuMSeoJJiaLBoudyguinaEGebreAKColvinPLSmithTLMurphyRCMishraNParksJS: Macrophage 12/15 lipoxygenase expression increases plasma and hepatic lipid levels and exacerbates atherosclerosis. *J Lipid Res*.2012;53(4) : 10.1194/jlr.M022723 686-95 10.1194/jlr.M022723 22279185PMC3307645
